# β-catenin-α-catenin and actomyosin signaling differentially regulate growth cone contours and axon undulation and branching of retinal ganglion cells *in situ*

**DOI:** 10.3389/fncel.2025.1572298

**Published:** 2025-07-22

**Authors:** Valerie Lew, Sukaynah Khetani, Simran Kaur, William Woodward, Sukmin Sandhu, Radhika Rawat, Tamira Elul

**Affiliations:** ^1^Department of Foundational Biomedical Sciences, College of Osteopathic Medicine, Touro University California, Vallejo, CA, United States; ^2^College of Osteopathic Medicine, Touro University Nevada, Henderson, NV, United States; ^3^Department of Molecular and Cellular Biology, University of California, Berkeley, Berkeley, CA, United States

**Keywords:** growth cone, adhesion, cytoskeleton, axon, Xenopus, retinal ganglion cells, beta-catenin, myosin II

## Abstract

**Introduction:**

Cadherin adhesive and actomyosin signaling are key cytomechanical cues required for neuronal circuit formation, but whether they function together to sculpt developing neurons is not known. Previously, we demonstrated that a β-catenin mutant (β-catNTERM) that disrupts binding of endogenous, full length β-catenin to α-catenin in the Cadherin adhesion complex, and a pharmacological inhibitor for actin regulator, non-muscle Myosin II (Blebbistatin), resulted in growth cones with fewer and more filopodia or filopodia-like protrusions than control growth cones of retinal ganglion cells (RGCs) in brains from *Xenopus laevis* embryos.

**Methods:**

Here, we assessed whether perturbation of β-catenin adhesive and Myosin II signaling specifically impacted additional, diverse yet interrelated, parameters of growth cone morphology and axon pathfinding, including two novel measures of growth cone contours.

**Results:**

Among other findings, we show that growth cones of individual RGCs expressing β-catenin NTERM have less complex contours (lower fractal dimension) and axons that are more undulatory than control growth cones and axons. In contrast, contours of Blebbistatin exposed growth cones are less concave (lower fractional concavity) and their axons extend more branches compared to control RGCs. In additional experiments, an α-catNTERM mutant and ROCK inhibitor phenocopied the specific effects of β-catNTERM and Blebbistatin on complexity and concavity of growth cone contours.

**Discussion:**

This data suggests that β-catenin-α-catenin and actomyosin interactions differentially regulate growth cone contours as well as axonal undulation and branching of RGCs in whole brains. Broadly, our results provide insight into cytomechanical mechanisms of neuronal circuit formation normally, and neuronal connectivity defects in human neurodevelopment disorders associated with mutations in Cadherin and β-catenin.

## Introduction

The cadherin cell–cell adhesion complex is an essential cyto-mechanical cue proposed to serve as a part of a ‘molecular clutch’ in developing axons, linking the actin cytoskeleton to the plasma membrane to exert traction on the substrate and drive growth cone advance (Bard et al., 2008). Numerous cell and developmental studies have shown that cadherins are required for normal growth cone morphology and axonal projections in a variety of developing neurons.

Cadherins modulate collapse and expansion of, as well as directional polarization of filopodia, balance between lamellipodia and filopodia, and extension of veils in, growth cones in neurons developing *in vitro* and *in vivo* (Yonekura et al., 2007; Payne et al., 1992; Schwabe et al., 2013; Polinsky et al., 2000). Cadherin adhesion factors are also required for proper fasciculation and targeting of axons in developing neurons in mice, chicken, and frog embryos (Mesías et al., 2023; Friedman et al., 2015; Treubert-Zimmermann et al., 2002; Stone and Sakaguchi, 1996). We additionally demonstrated that the N-terminal domain of β-catenin, that mediates binding to α- catenin in the Cadherin adhesive pathway, regulates the number of filopodia in growth cones, and dispersion of RGC axons in the late optic tract in *Xenopus laevis* tailbud stage embryos (Elul et al., 2022). Loureiro and Peifer (1998) also showed that the adhesive function of Armadillo (β-catenin) is needed for proper dispersion, projection and undulation of axons in Drosophila embryos, whereas another group demonstrated that stabilization of β-catenin by Wnt factor APC is required for proper projection of RGC axons in zebrafish (Paridaen et al., 2009). The importance of cadherin-catenin signaling in neuronal circuit formation is underscored by recent papers linking mutations in Cadherin and β-catenin with human neurodevelopmental disorders, including intellectual disabilities, autism and epilepsy (Lee et al., 2022; Accogli et al., 2019; Wickham et al., 2019).

A second major cyto-mechanical cue in developing neurons is actomyosin signaling, which is part of the growth cone molecular clutch mechanism described above and has also been shown experimentally to modulate physical force generated by filopodia and lamellipodia extension in growth cones of chicken dorsal root ganglion axons developing in culture (Sayyad et al., 2015). Application of Blebbistatin, a pharmacological inhibitor that prevents interactions between Myosin II and actin, or Y-27632, which inhibits Rho Kinase (ROCK) ability to phosphorylate Myosin Light Chain Kinase and Myosin Regulatory Light Chain Kinase, resulted in increased length and rate of extension of filopodia-like protrusions in their growth cones of chicken retina explant, medulla, or spinal cord neuronal cell cultures (Rösner et al., 2007). This was demonstrated to occur by attenuation of actomyosin interactions leading to growth of microtubule based neuritic extensions (Rösner et al., 2007). In a different study, application of Blebbistatin or Y-27632 also increased filopodial extension in the proximal axon in growth cones of axons in chicken dorsal root ganglion explants (Loudon et al., 2006). Growth cones in cultured neurons from superior cervical ganglions in Myosin II knockout mice also had smaller areas and increased rates of filopodial extension (Bridgman et al., 2001). In our previous study, we additionally demonstrated that application of a Myosin II inhibitor, Blebbistatin, increased numbers and length of filopodia-like protrusions in growth cones of GFP expressing RGC axons in whole brains of *Xenopus laevis* late tailbud stage embryos (Elul et al., 2022). Taken together, these studies suggest that non muscle Myosin II signaling is a negative regulator of filopodial or filopodia-like extension in growth cones of various developing neurons *in vitro* and *in situ*.

Myosin II has also been shown to be required for proper turning of axons in chicken DRG explants and for the ability of cranial motor axons to exit the midline and turn toward exit points in mice *in vivo* (Loudon et al., 2006; Murray et al., 2010).

Although these previous papers determined some functions for Cadherin-catenin and actomyosin signaling in developing neurons, we lack a comprehensive understanding of whether these two essential cyto-mechanical pathways regulate multiple, diverse yet interrelated, parameters of growth cones and axons that are important for and reflective of axon pathfinding *in vivo*. It also remains unclear whether these pathways act independently, together (as proposed by the molecular clutch hypothesis), or in some complex combinatoric manner, to sculpt developing neurons. In this study, we assessed whether perturbing β-catenin adhesive and Myosin II signaling impacted growth cone size, shape and contours, as well as axon undulation and branching, of individual RGCs in whole mount brains from tailbud stage *Xenopus laevis* embryos. To assess contours of the β-catenin mutant and Blebbistatin growth cones, we used two novel measurements - fractal dimension and fractional concavity – that, to our knowledge, have not previously been applied to analyze growth cones of any developing neuron. Among other findings, our results show that expression of a β-catNTERM mutant that should disrupt interactions between endogenous, full-length β-catenin and α-catenin, normally required for strong Cadherin cell–cell adhesion, resulted in less complex growth cones and more undulatory axons than control growth cones and axons. In contrast, inhibition of Myosin II by application of Blebbistatin induced growth cones with lower fractional concavity, and axons with increased number of branches relative to control RGCs. Additionally, an α-catNTERM mutant and ROCK inhibitor mimicked the effects of β-catNTERM and Blebbistatin on complexity and concavity of growth cone contours of RGCs *in situ*. These data suggest that β-catenin-α-catenin adhesive and actomyosin signaling differentially regulate growth cone morphology and axon pathfinding of RGCs in the *Xenopus laevis* vertebrate embryo model. More broadly, these results may also provide insight into cellular and mechanical mechanisms underlying formation of neuronal circuits normally and neuronal connectivity defects in human neurodevelopmental disorders associated with mutations in Cadherin and β-catenin.

## Materials and methods

### *Xenopus laevis* tadpoles

*Xenopus laevis* embryos were generated by natural mating of pairs of male and female frogs primed with Human Chorionic Gonadotrophin. Embryos were cultured in a 10% modified Ringer’s solution (MMR) and staged according to Nieuwkoop and Faber (1956) and Zahn et al. (2022). All animal experiments were performed at Touro University California and were approved by the Touro University California Institutional Animal Care and Use Committee.

### Lipofection of DNA plasmids

**Figure 1:**
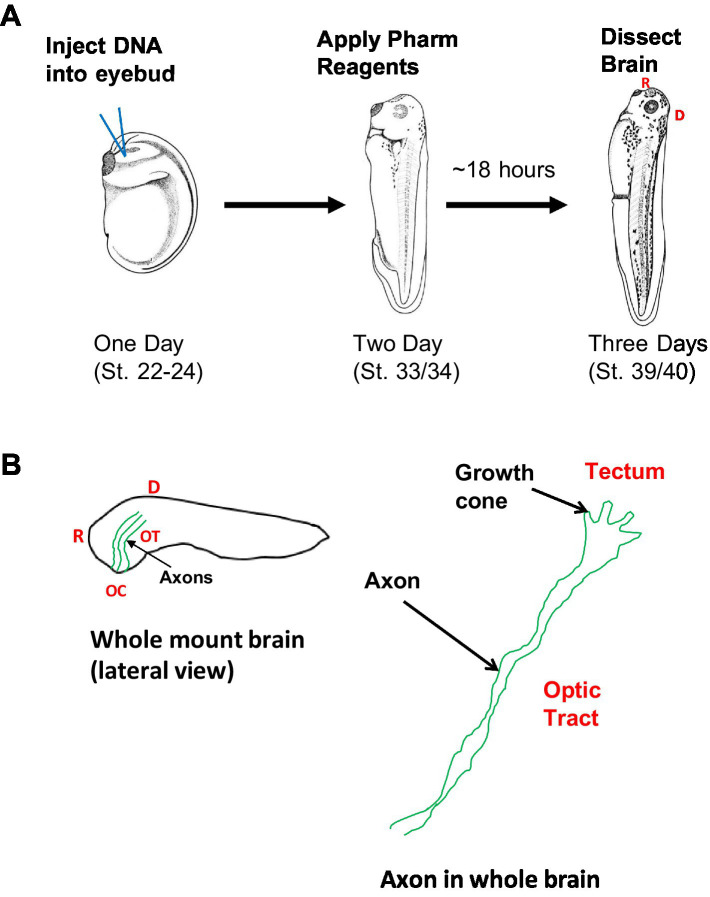
Schematic of experimental, imaging and image analysis approaches experimental approach **(A)**: pCS2-GFP, or pCS2-βcatNTERM-GFP, or pCS2-GFP together with pCS2-αcatNTERM were lipofected into developing eyebuds of one day old *Xenopus* embryos (stage 22; left, **A**). One day later, when RGC axons are beginning to enter the optic tract, some of the GFP lipofected embryos (stage 33) were placed in rearing solution containing pharmacological reagents (Blebbistatin or Y-27632) (middle, **A**). Approximately 18 h later, when tadpoles are at a developmental stage corresponding to when optic axons normally have reached the end of the optic tract, the control and experimental tadpoles (stage 39) were fixed and their brains were dissected and mounted (right, **A**). Imaging and analysis **(B)**: GFP control and experimental expressing growth cones and RGC axons were imaged in the optic tract of whole brains (left, **B**), and growth cones (size, shape and contours) and RGC axonal projections (linearity and branching) were analyzed (right, **B**).

We expressed the plasmids pCS2-GFP or pCS2-GFP-β-catNTERM in small numbers of retinal ganglion cells in *Xenopus laevis* embryos using a lipofection procedure (Dao et al., 2019; Jin et al., 2018; Wiley et al., 2008; Elul et al., 2003; Ohnuma et al., 2002; Holt et al., 1990). pCS2-GFP or pCS2-GFP-β-catNTERM (at a concentration > 1 μg/μl) were combined with a DOTAP lipofection reagent at a 1:3 weight to volume ratio. In other experiments, we mixed a pCS2-α-catNTERM plasmid with pCS2-GFP at a 1:1 weight ratio and then combined this mixture with DOTAP at a 1:3 DNA: DOTAP ratio (Wiley et al., 2008; Elul et al., 2003). We constructed the pCS2-α-catNTERM plasmid by subcloning the first 750 base pairs of chicken α- Ncatenin in a pCS2-αN-catenin plasmid into another pCS2 + plasmid using EcoR1 and XhoI [also see Sehgal et al. (1997)]. We injected 50–200 nL of these DNA-DOTAP solutions into eyebud primordia of one day old (stages 23–24) Xenopus embryos, as described previously (Dao et al., 2019; Jin et al., 2018; Wiley et al., 2008; Elul et al., 2003; Ohnuma et al., 2002; Holt et al., 1990; Figure 1A).

### Application of pharmacological reagent

Embryos lipofected with pCS2-GFP were reared in 0.1X Modified Mark’s Ringers (MMR) for approximately one more day at room temperature until they had developed to the late tailbud stage (developmental stages 33/34), which corresponds to when RGC axons normally enter into the optic tract. Some of the late tailbud stage pCS2-GFP lipofected embryos were then transferred to petri dishes containing 10 mls of 0.1XMMR bath solution with 10 μls of 1,000X Blebbistatin (Non-muscle Myosin II inhibitor, Sigma), or of 1,000X Rho Kinase Inhibitor (Y- 27632, Sigma) in vehicle [DMSO (Blebbistatin) or water (Y-27632)] added (Figure 1A). This resulted in a 10 μM final bath concentration for the pharmacological reagents, which was within the range of concentrations used in previous studies that applied pharmacological compounds to the bath of tissue preparations from *Xenopus laevis* tadpoles (Elul et al., 2022; Czesnik et al., 2007; Chien et al., 1993). In a previous study we showed that application of DMSO vehicle alone to *Xenopus* embryos containing GFP expressing RGC axons did not result in any axonal phenotype (Elul et al., 2022).

The tailbud stage embryos developed in the 0.1 X MMR bath solution with vehicle with pharmacological reagents for approximately 18 more hours at room temperature, until they had become young tadpoles (developmental stages 39–40; Figure 1A). These developmental stages correspond to when RGC axons normally reach the end of the optic tract and begin to enter into the optic tectum (Chien et al., 1993; Holt, 1989; Harris et al., 1987). Almost all tadpoles in Ringers’ solution containing vehicle with Blebbistatin (96%) or vehicle with ROCK inhibitor (100%), survived and developed to stages 39–40.

### Dissection of whole mount brains

When control (pCS2-GFP) and experimental (pCS2-GFP-β-catNTERM or pCS2-α-catNTERM expressing, or BLEB or Y-27632 exposed) tadpoles had reached developmental stages 39/40, they were then anesthetized in a 0.02% tricaine solution, and fixed in 4% Paraformaldehyde (PFA) for 1–2 h at room temperature. Following fixation, we rinsed the tadpoles three times in PBS. Fixed tadpoles were placed in a petri dish containing PBS, and their brains (forebrains and midbrains) were dissected using fine forceps (Dumont # 5). Tadpole brains were mounted lateral side up in PBS in a chamber containing a stack of two Avery notebook reinforcements on a glass slide sealed with a cover slip (Figure 1B).

### Imaging growth cones of optic axons in whole brains

Imaging of control and experimental GFP expressing axons in whole brains was performed with a Nikon Eclipse E800 widefield upright microscope equipped with epifluorescence (Mercury Arc illumination) and a motorized z-stage (Applied Scientific Instrumentation, MFC-2000), using a Nikon color camera (DS-5 M) with controller (DS-U1) driven by Nikon elements software (Version 3.1), or a Scion Corporation monochrome camera (CFW-1312 M) or Q-imaging Retiga monochrome camera (R1) driven by micro-manager software (Version 1.4 or Version 2.1). To best resolve individual RGC axons and growth cones in whole brains, we captured a z-series of 3–5 optical slices at 20X (Nikon Plan Apo 20x/0.75 DIC M) and 100X (Nikon Plan Apo VC 100X/1.40 Oil objective), respectively. These z-stacks were then manually reconstructed into a single image by tracing the visible component of axons and growth cones in individual slices in ImageJ (Version 1.41, NIH). All analysis for 20X images of axons was performed on these reconstructions with reference to the original images.

### Morphometric measurements

**Figure 2:**
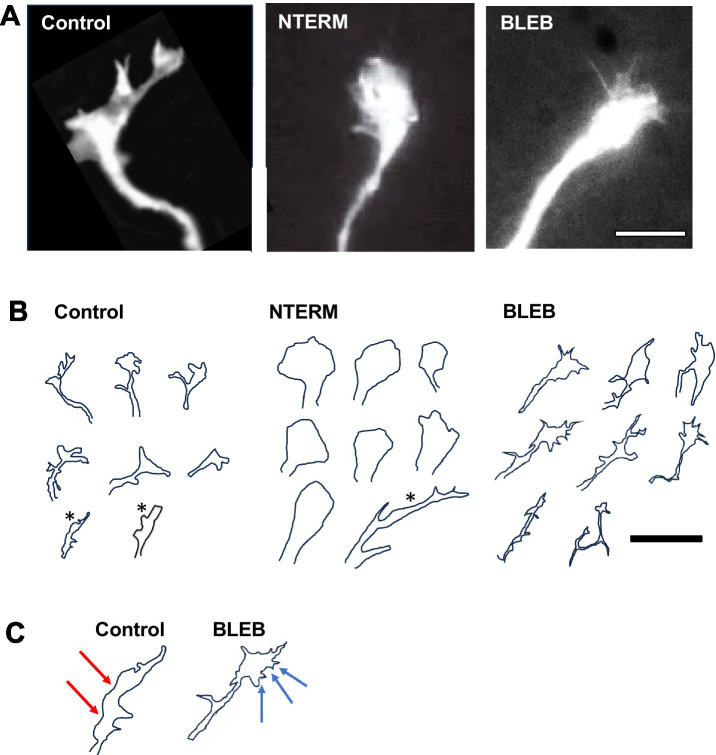
β-catNTERM and Blebbistatin modulate growth cones of RGC axons *in situ*. Representative microscopic images **(A)** and tracings **(B,C)** show differences between growth cones of control and experimental (β-catNTERM expressing and Blebbistatin exposed) GFP expressing growth cones in the optic tract of whole brains. Scale Bars: **(A)** 10 μm, **(B)** 15 μm.

Growth Cone Measurements: All quantitative analyses of growth cone morphology were made on reconstructions of 100X images captured of GFP expressing growth cones located in the mid to dorsal optic tract (Figure 2). In Image J (NIH version), we used the freehand tool to first outline individual growth cones including all protrusions, and then calculated *area and perimeter* of this outlined shape. We also measured the length and width of this outlined form for the growth cones. To obtain an *aspect ratio* for each growth cone, we divided the length of the growth cone by the width. *Circularity* of the growth cones was also calculated using the following formula (4*pi*area/perimeter^2). To calculate *fractal dimension* for growth cones, we used the box counting method (Balmages et al., 2021). For each growth cone, the border was traced and the tracing was superposed on grids of increasing box sixes (r, with a total of five different box sizes) in Microsoft PowerPoint. Boxes that contained any segment of the growth cone border were filled with red and counted (N). Using Excel, we plotted log N versus log (1/r) on an x-y scatter plot and fit the data points on a linear regression line. The slope of the linear regression line was recorded as the fractal dimension. To calculate *fractional concavity*, the border outline of each growth cone was imported into Image J software. Using the freehand tool in Image J, the total border length as well as the length of each visually identified concave segment of the border were measured. In Excel, the values of all the individual concave lengths were summed. This value was then divided by the total border length to find fractal concavity.

Axon Measurements: All quantitative analyses of RGC axon morphology were made on reconstructions of 20X images captured of GFP expressing axons located in the mid to dorsal optic tract. To assess axon undulation, we first superposed a 50 μm straight line on the terminal part of the axon, and then measured the total meandering length of the axon between the start and end points of the straight line. To obtain a measure of *rectilinearity*, we divided the meandering length of the axon by the length of the straight line (50 μm) (Wen et al., 2009; Bouquet et al., 2004). To quantify *branching*, we simply counted all the branches of any length that extended off the axon shaft.

### Statistical analysis

Images of growth cones and axons were independently measured by two or more researchers blind to the condition and their results were averaged. All data was stored in an Excel spreadsheet and means and standard errors [standard deviation divided by sqrt (n-1)] were calculated. Sample numbers are listed in the figure legends. Statistical significance of difference was determined using Students’ *t*-test (one tailed).

## Results

### β-catenin NTERM mutant and Blebbistatin impact size of RGC axonal growth cones *in situ*

In a previous paper, we showed that β-catenin interactions with α-catenin, and the actin regulator, non-muscle Myosin II, oppositely modulated numbers of growth cone filopodia (or filopodia like protrusions) in RGC axons in the optic tract of tailbud stage Xenopus embryos (Elul et al., 2022). To further investigate how these factors impact developing neuronal morphology, we again lipofected GFP, or a GFP-tagged β-catNTERM mutant (designed to competitively disrupt binding of endogenous, full-length β-catenin to α-catenin) into individual RGCs in eyebuds of one day old Xenopus embryos, or applied Blebbistatin, an inhibitor of non- muscle Myosin II, to embryos containing GFP expressing RGCs at a stage when their axons have just entered the optic tract (Figure 1). These control and experimental Xenopus embryos were then fixed at developmental stages 39/40, when RGC axons are in the mid to late optic tract (or have just arrived at the tectum), and their brains were dissected and mounted (Figure 1). Growth cones and axons of GFP expressing RGCs were imaged in the mid to late optic tract in the lateral diencephalon of these whole mount brains, and multiple parameters of growth cone morphology and axon pathfinding were analyzed from these images. We first describe the effects of the β-catenin N-terminal domain mutant and Blebbistatin Myosin II inhibitor on growth cone size (area and perimeter) of RGC axons, a feature previously shown to inversely correlate with axonal extension rates in Aplysia bag cell neurons *in vitro* (Ren and Suter, 2016).

**Figure 3:**
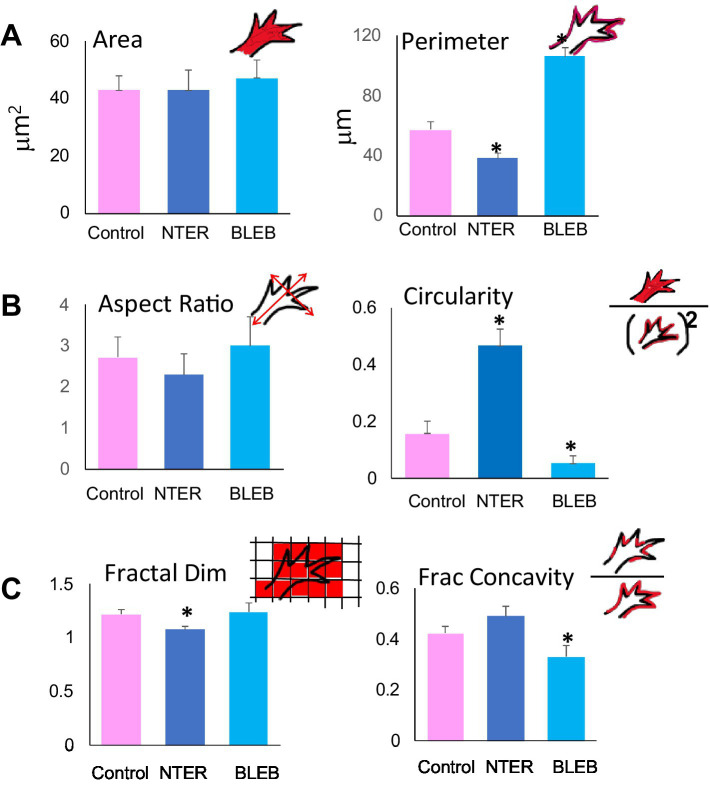
Quantitative analysis of control and experimental growth cones of RGC axons *in situ*. Quantification of morphometric parameters for control, β-catNTERM expressing and Blebbistatin exposed growth cones of RGCs in the optic tract confirms specific differences in size **(A)**, shape **(B)**, and contours **(C)** between control, β-catNTERM expressing and Blebbistatin exposed growth cones. Number Growth Cones Analyzed: Area and Perimeter: 14 Control, 13 β-catNTERM, 11 Blebbistatin. Circularity: 14 Control, 13 β-catNTERM, 11 Blebbistatin. Aspect Ratio: 14 Control, 13 β-catNTERM, 11 Blebbistatin. Fractal Dimension: 11 control, 11 β-catNTERM, 10 Blebbistatin. Fractional Concavity: 11 control, 11 β-catNTERM, 10 Blebbistatin.

Most (8/12) GFP expressing RGC control growth cones exhibited a moderately large size, with protrusions comparable in scale to the growth cone body control growth cones examined; Figures 2A,B. However, some (4/12) control growth cones appeared to lack protrusions and were significantly smaller than the other control growth cones (12 control growth cones examined, *, Figure 2B). β-catenin NTERM mutant expressing growth cones, most of which lacked any or had one filopodial protrusion, also showed variability in size, ranging from significantly smaller than to similar in size as to larger than growth cones of control RGC axons *in situ* (13 β-catNTERM growth cones, Figures 2A,B). In contrast, Blebbistatin exposed growth cones all had multiple fine protrusions and were comparable in size or slightly larger than control growth cones (19 Blebbistatin growth cones examined, Figures 2A–C). Measurements of areas of growth cones confirmed these differences in size between control and experimental growth cones. The average areas of β-catNTERM mutant expressing and Blebbistatin exposed growth cones were approximately the same as the area of control growth cones (Figure 3A; *p* > 0.05 for both).

We also assessed the perimeters of the control, β-catenin mutant expressing, and Blebbistatin exposed, growth cones of RGC axons in the optic tract. Control growth cones generally exhibited long perimeters, due to their protrusions (Figure 2). However, because most β-catNTERM growth cones lacked or had only one filopodial protrusions, their perimeters were significantly shorter than those of control growth cones (Figures 2A,B). In contrast to the β-catNTERM mutant growth cones, Blebbistatin exposed growth cones all had multiple, long thin protrusions, resulting in perimeters that were longer than those of control axons (Figures 2A,B).

Quantitative measurements showed that growth cones expressing the β-catNTERM mutant had an average perimeter that was 31% smaller than that of the control growth cones (*p* < 0.05; Figure 3A), whereas the mean perimeter of Blebbistatin exposed growth cones of RGC axons was 84% larger than that of control growth cones (*p* < 0.05; Figure 3B).

These data show that expression of the β-catNTERM mutant that disrupts interactions between α-catenin and β-catenin, and application of the Blebbistatin inhibitor for non-muscle Myosin II differentially alter the sizes of growth cones of RGC axons in the optic tract of *Xenopus laevis* tadpoles. Growth cones expressing the β-catNTERM mutant were reduced in perimeter whereas Blebbistatin exposed growth cones were significantly larger in perimeter than control growth cones of RGC axons *in situ*.

### β-catNTERM and Blebbistatin also modify shape of RGC growth cones in the optic tract

As noted above, we previously showed that growth cones of RGCs expressing the β-catenin mutant or exposed to Blebbistatin had fewer and more filopodial protrusions than control RGC growth cones, respectively (Elul et al., 2022). However, whether these changes in numbers of filopodia in β-catNTERM and Blebbistatin growth cones also led to specific alterations in the overall shape (in addition to size) of the growth cones was not determined. To explore this question, we next examined whether the β-catenin N-terminal domain mutant and Blebbistatin reagent differentially modified the elongation and/or roundness of growth cones of RGCs in whole brains from late tailbud stage *Xenopus* embryos (see methods; Elul et al., 2022; Sohal et al., 2017; Lakhani et al., 2016).

Most control, GFP expressing growth cones that we examined resembled hands with fingers splayed in different directions and appeared somewhat elongated (eight of 12 control growth cones; Figures 2A,B). However, the four control growth cones that lacked protrusions and were smaller than the other control growth cones were also significantly more narrow (*, Figure 2B). In contrast, eight of 12 β-catenin NTERM mutant expressing growth cones resembled hands closed into fists and the remaining β-cat mutant growth cones were branched and very elongated; Accordingly, β-catNTERM mutant growth cones showed varying degrees of elongation from wider than to more narrow than control growth cones (Figures 2A,B). In addition, all 11 Blebbistatin exposed growth cones resembled hands with fingers extending in the same direction, and consequently, appeared a bit more than elongated than control growth cones (Figures 2A,B). Confirming these observations, the mean aspect ratio (L/W) for all β-catNTERM mutant expressing growth cones was ~ 16% less than that of control growth cones (*p* > 0.05, Figure 3B), whereas Blebbistatin exposed growth cones had an aspect ratio 5% greater than that of control growth cones (*p* > 0.05, Figure 3B).

Although elongation describes some features of the shape of the RGC growth cones in the optic tract, it does not fully account for their complex morphology *in situ*. Thus, we next examined whether a second shape feature – circularity - was also altered in the experimental growth cones (Elul et al., 2022; Sohal et al., 2017). To establish baseline measures, we first analyzed the circularity of the control, GFP expressing, growth cones. Control growth cones had irregular borders and elongated shapes and accordingly, did not appear very circular (Figures 2A,B). In contrast, most of the β-catNTERM mutant expressing growth cones (except for three branched β-catNTERM mutant growth cones) that we examined were significantly more round than the control growth cones (Figures 2A,B). Conversely, Blebbistatin exposed RGC growth cones appeared even more irregular and less round than the control growth cones. Quantification showed that for β-catNTERM expressing growth cones, the average circularity was 113% greater than that calculated for growth cones of control RGC axons (*p* < 0.05; Figure 3B), whereas for growth cones from embryos exposed to Blebbistatin, the mean circularity index was 68% less than that of control growth cones *in situ* (*p* < 0.05; Figure 3B).

These results indicate that the β-catNTERM domain mutant that disrupts interactions of endogenous β-catenin and α-catenin and the Blebbistatin inhibitor of Myosin II differentially altered the roundness of growth cones of RGC axons in the optic tract. Most growth cones expressing the N-terminal domain of β-catenin were more circular or round than control growth cones of RGCs axons *in sit*u, whereas growth cones of RGCs in embryos exposed to Blebbistatin were significantly less round than those of control RGC axons.

### β-cat mutant and Blebbistatin growth cones show differential changes in their contours

In addition to the β-catenin mutant and Blebbistatin inhibitor altering the size and shape of the RGC growth cones, these perturbations of adhesive and cytoskeletal signaling may also change the contours of the growth cones of RGC axons *in situ*. To our knowledge, growth cone contours have not been assessed in a developing neuron in any prior study. To measure contours of control and experimental growth cones, we calculated the fractal dimension (roughness or rate of change of detail with scale), a measure previously used to quantify contours of non-neuronal cell types with complex morphologies such as killifish pigment cells, starfish coelomocytes and cancer cells (Karetin, 2021; Uppal et al., 2010). For a second measurement of growth cone contours, fractional concavity (percentage of the contour that is concave) was also determined. In a prior study, fractal dimension and fractional concavity applied together were able to effectively differentiate contours of malignant from benign masses in mammogram images of breast tissue (Rangayyan and Nguyen, 2007).

Contours of control, GFP expressing control growth cones in the optic tract of whole brains were rough with irregular undulations (Figures 2A,B). Even the four GFP growth cones that lacked protrusions and were smaller and narrower than the other control growth cones displayed relatively rough contours (*, Figure 2B). However, the contours of all β-catNTERM mutant expressing growth cones (including the branched β-catNTERM mutant growth cones) were more smooth than those of control growth cones (compare left and middle images in Figures 2A,B). Conversely, contours of Blebbistatin exposed growth cones appeared similarly rough as, or more rough than, the growth cones of control RGC axons (Figure 2). Measurements showed that the mean fractal dimension determined for β-catNTERM mutant expressing growth cones was 13.6% lower than that of control growth cones (*p* < 0.05; Figure 3C). However, for Blebbistatin exposed growth cones, the average fractal dimension was not significantly different than that of control growth cones (*p* > 0.05).

To further determine whether the contours of β-catNTERM mutant and Blebbistatin exposed growth cones differed from those of control GFP expressing growth cones and from each other, we also measured their fractional concavity (the relative portion of the growth cone contour that is concave). Compared to control GFP expressing growth cones, β-catenin mutant expressing growth cones had similarly long regions of concavity, whereas Blebbistatin exposed growth cones displayed shorter regions of concavity (arrows, Figure 2C). Confirming these observations, for β-catNTERM expressing growth cones, we calculated a mean fractional concavity similar to that of control growth cones (*p* > 0.05; Figure 3C), whereas the mean fractional concavity for Blebbistatin exposed growth cones was 24% less than the mean fractional concavity determined for control growth cones (*p* < 0.005; Figure 3C).

This analysis shows that the β-catenin NTERM mutant and the Blebbistatin inhibitor differentially modify contours of growth cones of RGC axons in whole brains from *Xenopus* tailbud stage embryos. Growth cones that expressed the β-catenin N-terminal domain mutant were less complex (had lower fractal dimension) than, but of similar concavity (same fractional concavity) as, control growth cones of GFP expressing RGC axons in the optic tract. In contrast, Blebbistatin exposed growth cones had similar fractal dimension but lower fractional concavity than control growth cones of RGC axons in whole brains.

### Undulation of RGC axons in the optic tract are also modified by β-catNTERM and BLEB

Thus far we have shown that expression of the β-catenin N-terminal domain mutant resulted in growth cones in RGCs in *Xenopus laevis* embryos that have smaller perimeters, increased circularity and less complex contours, whereas application of Blebbistatin induced growth cones with larger perimeters, decreased circularity and decreased contour concavity, compared to control growth cones in RGCs in the optic tract. These findings extend our previously published data demonstrating that the β-catNTERM mutant and Blebbistain decreased and increased the number of filopodia or filopodia-like protrusions in growth cones of RGCs *in situ*, respectively (Elul et al., 2022). However, in our earlier paper, we also showed that expression of the β-catNTERM mutant increased dispersion of RGC axons compared to control RGC axons in the optic tract (Elul et al., 2022). To further explore how the specific alterations in growth cone morphology induced by the β-catNTERM mutant and Blebbistatin inhibitor impact RGC axonal projections, we next investigated whether these perturbations changed the fine scale undulation or tortuosity of individual RGC axons in the optic tract. In a previous paper on the developing chick spinal cord, undulation of axons was shown to correlate with the degree of external mechanical stretch applied to the tissue (Hao and Shreiber, 2007).

**Figure 4:**
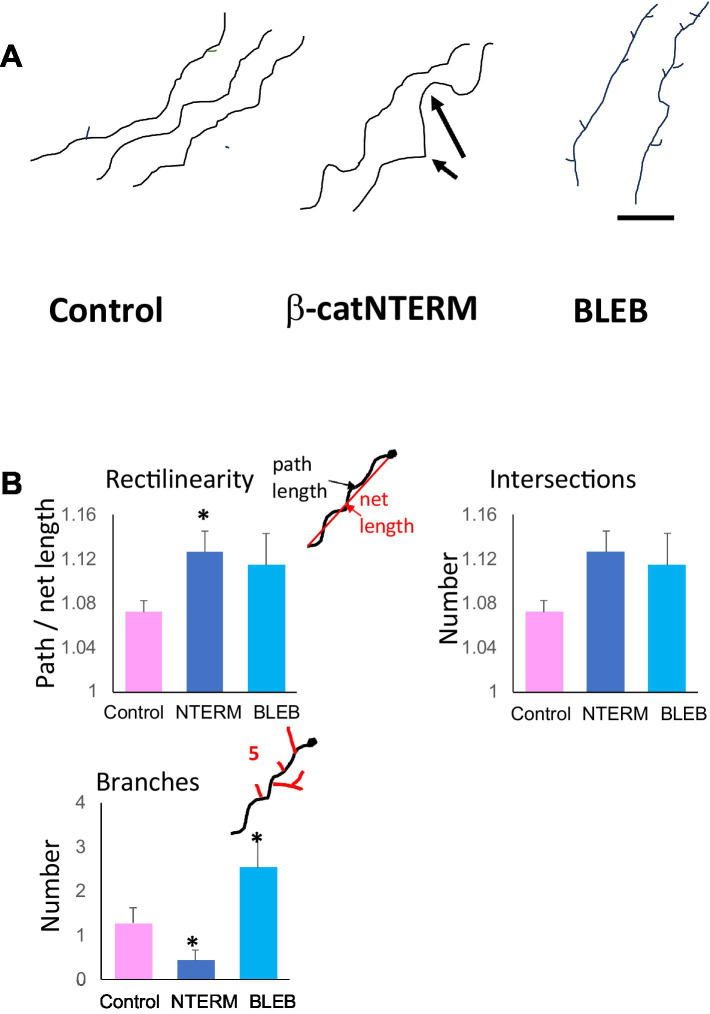
β-catNTERM and Blebbistatin regulate RGC axonal projections in the optic tract of whole brains. Representative tracings of axonal projections **(A)**, and plots of morphometric measurements **(B)** show differences between control, β-catNTERM and Blebbistatin exposed RGC axons in the optic tract of whole brains from late tailbud stage *Xenopus* embryos. Scale Bar: A - 50 μm. Number axons analyzed: Rectilinearity- 16 control, 9 β-catNTERM, 13 Blebbistatin; Number Branches - 14 control, 9 β-catNTERM, 13 Blebbistatin.

Most control, GFP expressing RGC axons that we examined displayed small amplitude undulations throughout their trajectories in the optic tract (Figure 4A). However, many RGC axons expressing the β-catNTERM mutant exhibited both a greater number of and larger undulations than control axons (arrows, middle panel, Figure 4A). Blebbistatin exposed axons also showed increased fine-scale waviness compared to control axons, though not to the same degree, or as consistently, as the β-catenin mutant expressing axons (right panel, Figure 4A). As an example, the Blebbistatin axon trace on the far right of Figure 4A displays a single large undulation but lacks the smaller undulations that we observed in the β-catNTERM expressing axons (right panel, Figure 4A). To quantify the waviness of the axons, we measured the rectilinearity ratio, defined as the path (actual) length of the axon divided by the straight line (end-to-end) length [see Methods; Wen et al. (2009)]. This analysis showed that the average rectilinearity ratio for β-cat mutant axons was 5.6% greater than that of control growth cones (*p* < 0.05; Figure 3B), whereas the difference between the average rectilinearity ratio of Blebbistatin exposed and control axons was not statistically significant (*p* > 0.05).

As a second assessment for waviness of control and experimental RGC axons in the optic tract, we also measured the number of intersections of the axons with a straight line 50 μm in length superposed on the terminal segment of the axons. The mean number of intersections of β-catNTERM axons with the line was 77% greater than that of control axons. Similarly, for Blebbistatin exposed axons, we calculated a mean number of intersections that was 75% greater than that of the control axons. However, although the mean number of intersections of both β-catNTERM and Blebbistatin axons with the line was much larger than that of control RGC axons, the differences were not statistically significant, due to the small absolute values and the relatively large variabilities in this parameter.

Thus, in addition to expression of the β-catenin NTERM mutant that disrupts interactions between endogenous, full-length β-catenin and α-catenin modifying growth cone morphology, it also alters the projections of RGC axons in the optic tract. β-catNTERM significantly increased the tortuosity of RGC axons relative to control optic axons in the optic tract in whole brains.

### β-catNTERM and Blebbistatin oppositely modify branching in axons of RGCs in the tract

When RGC axons reach the end of the optic tract and enter the tectal midbrain, they extend branches along the axon shaft. At later stages of development, when RGC axons enter the tectal midbrain they will elaborate terminal arbors to make synaptic connections with interneurons in the tectum. In previously published papers, we showed that expression of the β-catenin N-terminal domain mutant used in this study decreased the number of branches in RGC axonal arbors in dorsal tecta of older, living *Xenopus laevis* tadpoles at developmental stages 45–46 (Elul et al., 2003; Wiley et al., 2008). In this study, we explored whether expression of β-catNTERM and application of Blebbistain also modified the number of axon shaft branches extended by the RGCs in the optic tract of younger, late tailbud stage *Xenopus* embryos at developmental stages 39/40.

Similar to the previous findings of other researchers (Harris et al., 1987), we observed that most control RGC axons extended few branches in the optic tract of whole brains from late tailbud stage *Xenopus* embryos (Figure 4A). In our images, control, GFP expressing axons in the optic tract/tecum had between 0 and 2 lateral branches. However, RGC axons expressing the β-catNTERM mutant extended even fewer branches than control RGC axons in the optic tract whereas application of the Blebbistatin inhibitor resulted in GFP expressing RGC axons with significantly increased numbers of lateral branches compared to controls (Figure 4A). All β-cat mutant expressing axons that we examined displayed no secondary branches whereas Blebbistatin exposed axons extended 2–7 branches along the axon shaft (compare representative axon tracings in Figure 4A). The mean number of branches for β-catNTERM mutant expressing axons was 70% less than controls, whereas the mean number of branches for BLEB exposed axons was 94% greater than, that of controls (*p* < 0.05 for both; Figure 4B).

These data show that the β-cat NTERM mutant and Myosin II inhibitor, Blebbistatin, oppositely modulate branching in RGC axons in tailbud stage *Xenopus* embryos. Expression of the β-catNTERM mutant decreased branching in RGC axons whereas Blebbistatin increased the number and length of branches extended by RGC axons in the optic tract.

### α-catenin mutant and ROCK inhibitor phenocopy β-catNTERM and BLEB growth cones

The data presented here indicate that the β-catNTERM mutant and the Myosin II inhibitor, Blebbistatin, differentially modified multiple parameters of growth cone morphology and axonal projections of RGCs in *Xenopus* embryos. To determine the molecular mechanisms by which β-catNTERM and Blebbistatin alter morphology of RGC growth cones and axons, we next carried out experiments in which we perturbed the activity of their interacting or regulatory factors. To confirm that the β-catNTERM mutant disrupts interactions between endogenous, full-length β-catenin and α-catenin, we constructed an N-terminal domain fragment of α-catenin (α-catNTERM) that contains the β-catenin binding domain but lacks the C-terminal region of α- catenin that normally mediates or modulates its’ interaction with actin. When overexpressed in cells, α-catNTERM should, like β-catNTERM, competitively disrupt the interactions between endogenous, full length α-catenin and β-catenin, thereby impairing Cadherin mediated cell to cell adhesion. In a prior study, expression of a similar α-catNTERM mutant caused tissue disaggregation in younger *Xenopus* embryos, and in explants taken from the embryos (Sehgal et al., 1997). To confirm that Blebbistatin functions by inhibiting the activity of non-muscle Myosin II, we applied an inhibitor of Rho Kinase (Y-27632) to tailbud stage *Xenopus* embryos containing GFP expressing RGC axons. Rho Kinase normally phosphorylates Myosin Regulatory Light Chain Kinase and Myosin Light Chain Kinase, which in turn promote Myosin II ATPase activity. Because the β-catNTERM mutant and Blebbistatin inhibitor differentially modified both the complexity and concavity of growth cone contours, we examined whether the α-catNTERM mutant and ROCK inhibitor phenocopied these specific effects of β-catNTERM and Blebbistatin on the contours of growth cones of RGCs in the optic tract.

**Figure 5:**
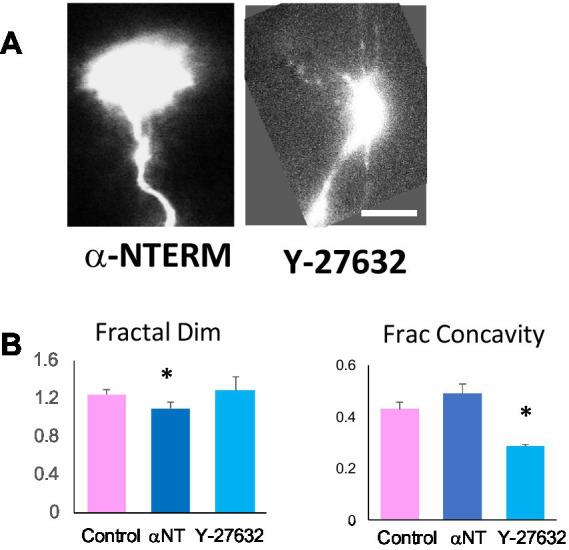
α-catNTERM mutant and ROCK inhibitor phenocopy β-catNTERM and Blebbistatin effects on growth cone contours. Representative images of α-catNTERM and Y-27632 exposed growth cones **(A)**. Scale Bar: A- 5 μm. Plots of morphometric measurements of contours show differences between control, α- catNTERM and ROCK inhibitor exposed growth cones **(B)**. Number growth cones analyzed for fractal dimension and fractional concavity: 11 control, 6 α-catNTERM, 6 Y-27632.

These experiments showed that a significant fraction of growth cones expressing the α-catNTERM mutant lacked protrusions and appeared similar to β-catNTERM expressing growth cones that lacked protrusions (Figure 5A; 4/6). The remaining α-catNTERM growth cones appeared long and branched, similar to the β-catNTERM growth cones that were branched (see description above). To quantitatively assess the form of α-catNTERM mutant expressing growth cones, we measured the fractal dimension and fractional concavity of the contours. This analysis showed that, similar to β-catNTERM expressing growth cones, contours of α-catNTERM expressing growth cones had lower fractal dimensions than control GFP- expressing growth cones. The mean fractal dimension of the α-catNTERM expressing growth cones was 11% lower than that of controls (*p* < 0.05). Further measurements showed that the mean fractional concavity of α-catNTERM expressing growth cones was similar to that of controls, (*p* > 0.05), again mimicking the phenotype of the β-catNTERM expressing growth cones.

In contrast to α-catNTERM expressing growth cones, growth cones of RGCs in embryos exposed to the ROCK inhibitor Y-27632 had more and/or longer protrusions than control growth cones (Figure 5A). Phenocopying the Blebbistatin exposed growth cones, quantitative measurements showed that the mean fractal dimension of the contours of Y-27632 exposed growth cones were similar to those of controls (*p* > 0.05). Moreover, similar to Blebbistatin growth cones, ROCK inhibitor growth cones showed shorter regions of concavity, which was reflected in lower measurements of fractional concavity compared to controls. The mean fractional concavity for Y-27632 treated growth cones was 33% smaller than that of control growth cones (p < 0.05).

This data shows that expression of the α-catNTERM mutant that disrupts interactions between endogenous full length α-catenin and β-catenin resulted in growth cones that were less complex (lower fractal dimension) but had similar fractional concavity as control growth cones, similar to the β-catNTERM mutant expressing growth cones. In contrast, ROCK inhibitor (Y- 27632) exposed growth cones had similar fractal dimension but lower fractional concavity compared to control growth cones, mimicking the effects of Blebbistatin on the growth cones of RGC axons in the optic tract.

## Discussion

In this paper, we applied morphometric measurements, including two novel variables of growth cone contours - fractal dimension and fractional concavity- to study the relative roles of cadherin cell–cell adhesive and actomyosin signaling on growth cone morphology and axonal projections of RGCs in the optic tract of whole mount brains of *Xenopus laevis* frog embryos. Our results show that RGC growth cones expressing a β-catNTERM mutant that disrupts interactions between endogenous, full-length β-catenin and α-catenin in the Cadherin adhesion pathway exhibited growth cones with less complex contours and more undulatory axons than control growth cones and axons *in situ*. In contrast, growth cones exposed to the non-muscle Myosin II inhibitor, Blebbistatin, displayed greater fractional concavity in their contours and increased axon branching relative to control RGCs in the optic tract. In addition, an α- catNTERM mutant and Rho Kinase inhibitor phenocopied the effects of β-catNTERM and Blebbistatin on growth cone contour complexity and concavity. These data suggest that β-catenin-α-catenin and actomyosin signaling interactions employ different mechanisms to regulate growth cone contours, and undulation and branching of RGC axons in late tailbud stage embryos of *Xenopus laevis*. As no previous scientific literature has determined the role of β-catenin or α-catenin in growth cone morphology, these findings provide significant new insight into the role of cadherin mediated adhesion signaling in shaping growth cone structure and guiding neuronal circuit development.

### Novel measurements for growth cone contours: fractal dimension and fractional concavity

To determine the specific functions of the β-catenin N-terminal domain and non-muscle Myosin II in modulating the contours of growth cones of RGCs *in situ*, we employed morphometric measurements of fractal dimension and fractional concavity. To our knowledge, these metrics have not previously been used to quantify growth cone contours, nor have prior studies used any other metrics to quantify growth cone contours. In earlier reports, fractal dimension was used to assess complexity of contours of non-neuronal cells such as killifish pigment cells, starfish coelomocytes, and cancer cells *in vitro* (Karetin, 2021; Uppal et al., 2010). Additionally, fractal dimension and fractional concavity were applied together to effectively differentiate contours of benign from malignant masses in mammogram images of breast tissues (Rangayyan and Nguyen, 2007). An earlier study also used fractal dimension to measure structural differentiation and branching of embryonic ventral spinal cord neurons on different substrates astrocytes versus poly (D-lysine, PDL) (Schaffner and Ghesquiere, 2001). In that paper, fractal dimension was similar for neurons on astrocytes and PDL, initially increasing, followed by a decrease. This higher fractal dimension value initially was qualitatively associated with expansive lamellipodia on the neuronal cell bodies grown on PDL (Schaffner and Ghesquiere, 2001). Fractal dimension has also been used to measure the relative straightness or undulation of axons of embryonic frog and chick neurons in culture (Katz, 1985). In the future, fractal dimension and fractional concavity could serve as useful morphometric tools to assess contours of growth cones of neurons following molecular and mechanical perturbations in other developmental neurobiology studies.

**Figure 6:**
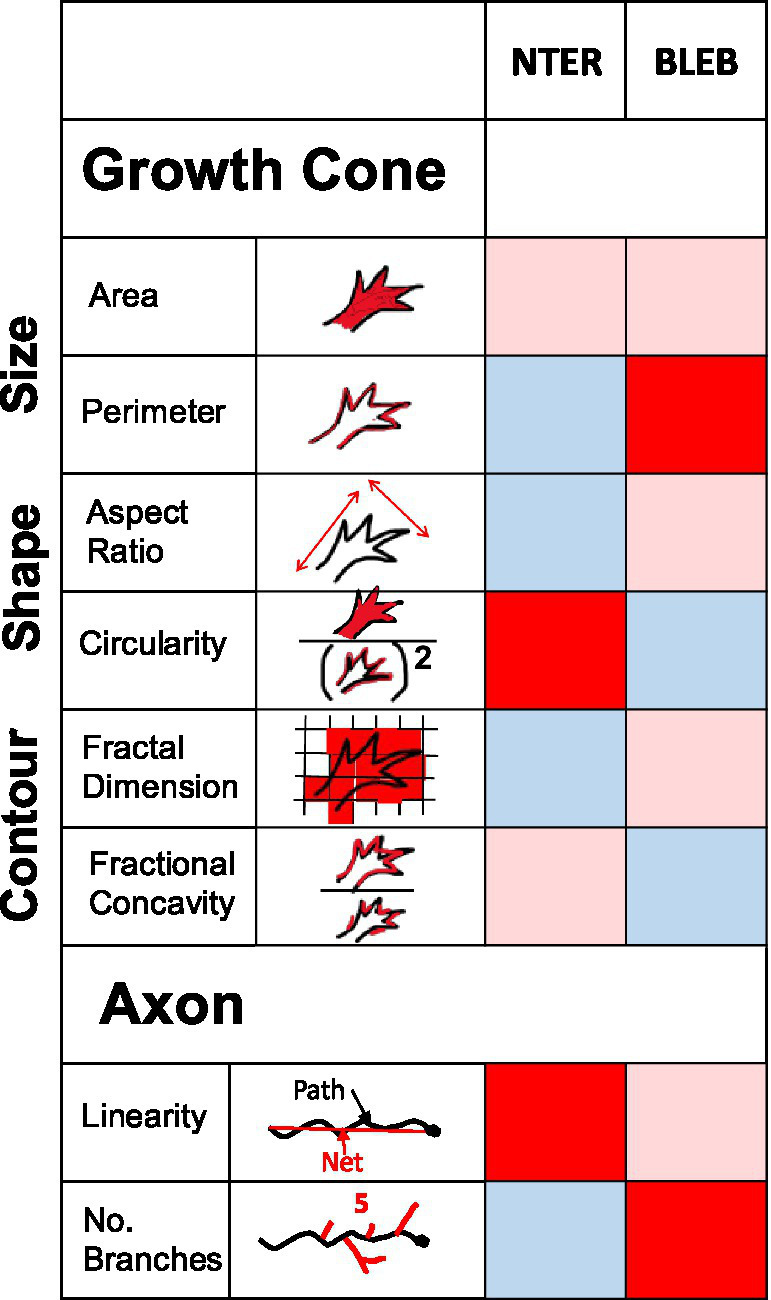
Summary of differential effects of β-catNTERM and Blebbistatin on RGC growth cones and axons *in situ*. Heat map table visualizes effects of β-catNTERM and Blebbistain on RGC growth cone and axon morphological parameters. Light pink shading in cell indicates no significant change from control, dark red shading in cell indicates a significant increase in morphological parameter value compared to controls, and light blue shading in cell indicates a significant decrease in morphological parameter compared to controls.

As summarized in the heat map in Figure 6, our findings reveal distinct changes in growth cone morphology across conditions. β-catenin NTERM mutant expressing growth cones exhibited reductions in mean perimeter, aspect ratio, and fractal dimension, alongside an increase in circularity. Conversely, Blebbistatin treated growth cones showed increased average perimeter and reduced circularity and fractional concavity, relative to control RGC growth cones in whole mount brains. The inclusion of fractal dimension and fractional concavity as novel morphometric metrics was critical to distinguishing these contour changes. These parameters offer insight into structural complexity and indentation patterns that are not captured by traditional measures such as growth cone area or aspect ratio. Together, our results suggest that the β-catenin N-terminal domain and Myosin II signaling distinctly regulate the geometry and contour dynamics of RGC growth cones *in situ*. To further interpret the biological significance of these measurements, it would be valuable to assess the statistical relationships among size, shape and contour parameters among both control and experimentally perturbed growth cones (Chitsaz et al., 2015). For instance, some parameters may show strong positive or negative correlations with each other in control conditions, but these relationships may break down or change in mutant or chemically treated neurons. However, as fractal dimension is a nonlinear metric, it may not correlate predictably with linear parameters such as area or aspect ratio. A prior study of growth cones of wild type spinal cord motoneurons *in vitro* assessed number of processes and area and shape of the growth cone convex hull and contour, concluding that parameters of the growth cone contour correlated with, and therefore, could serve as a proxy for quantification of the number of growth cone processes in control growth cone (Kawa et al., 1998).

Fractal dimension quantifies growth cone contour complexity by measuring how detail changes with scale, offering information distinct from that of size or shape. This is comparable to Sholl analysis, a commonly used method to assess arbor complexity in developing neurons (Marshak et al., 2012). While Sholl analysis evaluates branch density across concentric circles centered on the soma, fractal analysis assesses growth cone contour patterns across different size grids (Binley et al., 2014). To measure fractal dimension of growth cone contours, grids with different box dimensions were applied over a tracing of each growth cone contour, and the number of boxes that contained a piece of the contour was determined for each grid size. A log–log plot of number of boxes versus the inverse of box size was generated, and the slope of the line of this plot corresponded to the fractal dimension (also called box counting dimension). In Sholl analysis, a similar log–log plot can be used to relate the number of branch intersections with circle radius, and the slope of the rising phase of this plot is closely related to fractal dimension (Ascoli, 2002; Wen et al., 2009). This parallel underscores the utility of fractal analysis as a robust and quantitative approach to studying neuronal morphology.

### β-catenin-α-catenin signaling regulates growth cone morphology and axonal projections of RGCs *in situ*

Expression of the β-catenin N-terminal domain mutant - containing the α-catenin but lacking the Cadherin binding region - resulted in growth cones that were smaller, more round, and less complex (lower fractal dimension) than growth cones of control, GFP expressing RGCs in the optic tract of late tailbud stage embryos (Figure 6). Similarly, growth cones expressing a complementary α-catenin N-terminal domain mutant that contains the β-catenin binding site but lacks the actin interaction region also exhibited lower complexity than control growth cones.

Overexpression of either N-terminal domain is expected to disrupt interactions between endogenous, full-length β-catenin and α-catenin, that normally link Cadherin to actin to establish strong cell–cell adhesion. Accordingly, these results suggest that β-catenin-α-catenin adhesive signaling normally functions to increase perimeter, decrease roundness, and increase contour complexity of growth cones of RGC axons in the optic tract. These results extend our previous finding showing that the β-catenin N-terminal domain that contains the α-catenin interaction site is required to promote growth cone filopodia in RGCs in the optic tract of brains from *Xenopus* tadpoles (Elul et al., 2022), by both providing additional description of the altered morphology of the mutant expressing growth cones, and evidence supporting the notion that the β-catenin N- terminal domain regulates growth cone morphology by binding to α-catenin. To our knowledge, no prior studies have implicated either β-catenin or α-catenin in the regulation of growth cone.

morphology or filopodial dynamics in any developing neuronal system. However, the adhesive domain of Cadherin (that binds to β-catenin) was shown to be required for normal expansion of growth cones of R7 afferents in Drosophila (Yonekura et al., 2007). More broadly, our data also support results from other studies demonstrating that Cadherin function is required for normal growth cone shape, and filopodial number and direction, in a variety of developing neurons in different species (Yonekura et al., 2007; Payne et al., 1992; Schwabe et al., 2013; Polinsky et al., 2000). The absence of filopodia and reduced morphological complexity in β-catNTERM expressing RGC growth cones bears resemblance to the phenotype observed in RGCs in *Xenopus* embryos exposed to Cytochalasin B, an actin polymerization inhibitor (Chien et al., 1993). This parallel suggests that β-catenin-α-catenin signaling may regulate growth cone morphology by promoting actin polymerization, via a direct or indirect interaction of α-catenin with the actin cytoskeleton. Interestingly, the β-catenin mutant growth cones described here also resemble those seen in *Xenopus* embryos where the retina was removed and the axons were detached from their cell bodies, resulting in RGC growth cones with shorter, broader filopodia (See Figure 9 in Harris et al., 1987). This further supports the idea that β-catenin-α-catenin interactions may influence growth cone shape by modulating substrate traction and mechanical tension along the axon (Thoumine, 2008; Bard et al., 2008).

The data we present here also indicate that axons of RGCs expressing the β-catenin NTERM domain mutant are more undulatory than control RGC axons in the optic tract of brains from *Xenopus* embryos (Figure 6). This suggests that interactions of β-catenin with α-catenin in the Cadherin adhesive pathway are additionally required to inhibit undulation of RGC axons in the optic tract of *Xenopus laevis* embryos. This finding aligns with previous work by Loureiro and Peifer (1998) that demonstrated that disruption of the adhesive function of Armadillo (β-catenin) led to increased axon undulation in Drosophila embryos. Additionally, our results expand upon our earlier finding that the β-catenin N-terminal domain mutant increased dispersion of small groups of RGC axons in the optic tract (Elul et al., 2022), and support other papers demonstrating that perturbation of Cadherin function disrupts normal fasciculation of axons in various developing neuronal systems (Mesías et al., 2023; Friedman et al., 2015; Treubert-Zimmermann et al., 2002; Stone and Sakaguchi, 1996), or identifying a role for canonical Wnt/β-catenin signaling in axon pathfinding (Zuñiga et al., 2023; Paridaen et al., 2009). However, the precise relationship between undulation of individual axons and fasciculation of a group of axons has not been determined in any prior study. In another study, degree of axonal undulation was shown to inversely correlate with the magnitude of mechanical strain applied to developing spinal cords in chicken embryos (Hao and Shreiber, 2007), further suggesting that β-catenin and α-catenin interactions may also modulate the mechanical properties of the axons of RGCs. Axonal undulation has also been correlated with spatial efficiency of axonal navigation (Wen et al., 2009), and been observed to be increased in nervous system disorders, such as diabetic peripheral neuropathy and normal pressure hydrocephalus (Edwards et al., 2014).

We also demonstrated that expression of the β-catenin N-terminal domain mutant decreased the number of branches extended by RGC axons in the late optic tract/tectum in brains from late tailbud stage embryos of *Xenopus laevis* (developmental stages 39/40; Figure 6).

Previous timelapse imaging by Harris et al. (1987) demonstrated that RGC axons initiate branching in the late optic tract upon entry into the tectum by extending lateral branches along the axon shaft (Harris et al., 1987). Future studies using timelapse imaging of β-catNTERM expressing RGC axons could clarify whether β-catenin and α-catenin interactions specifically regulate the rate of branch extension and retraction. We previously showed that the β-catNTERM mutant used in this study also decreased the number of branches in RGC axonal arbors in dorsal tectal midbrains of older stage *Xenopus laevis* tadpoles (developmental stages 45/46; Elul et al., 2003). Taken together, this suggests that interactions of β-catenin with α- catenin in the Cadherin adhesion complex are required to promote branching of RGCs at two different developmental stages in *Xenopus laevis* embryos and tadpoles. A prior study described the development of topography of RGC axonal arbors between the late tailbud embryo and mid tadpole stages of *Xenopus laevis* in wholemounts and sections of fixed brains (Sakaguchi and Murphey, 1985). However, the dynamic progression of branching of RGC axons in living brains between the late tailbud and mid tadpole stages has not been assessed. Accordingly, we currently do not know whether or how the initial lateral branching of RGC axons develops into more mature terminal arborization of these axons and cannot predict how β-catenin interactions with α-catenin might regulate this morphogenetic process (Del Rio et al., 2023).

### Non-muscle myosin II also modulates growth cones and axons in RGC axons *in situ*

Our results also show that treatment of embryos with non-muscle Myosin II inhibitor, Blebbistatin, altered the morphology of growth cones of RGC axons in whole brains in a different manner than the β-catNTERM mutant (Figure 6). Growth cones of RGCs exposed to Blebbistatin had longer perimeters, were less circular, and had greater fractional concavity compared to control, GFP expressing, growth cones in the optic tract (Figure 6). Application of the ROCK inhibitor, Y-27632, similarly led to RGC axonal growth cones with increased fractional concavity *in situ*. These results suggest that activity of non-muscle Myosin II normally functions to decrease perimeter, increase circularity and decrease concavity of RGC growth cones *in situ*. This finding extends our previously published results showing that Myosin II functions to decrease numbers and length of filopodia-like protrusions in growth cones of RGCs in the optic tract of *Xenopus laevis* embryos (Elul et al., 2022). Consistent with our results, other groups also demonstrated that Blebbistatin or ROCK inhibitor treatment increased rate of extension and length of growth cone filopodia-like protrusions, or induced more filopodia and lamellipodia just distal to the growth cone, in embryonic chick DRG neurons *in vitro* (Rösner et al., 2007; Loudon et al., 2006). Additionally, neurons cultured from superior cervical ganglions of Myosin IIB knock-out mice, displayed growth cones with smaller areas and more frequent filopodial-like protrusions around the growth cone (Bridgman et al., 2001). Previous studies also demonstrated that inhibiting actomyosin interactions using Blebbistatin or the ROCK inhibitor Y-27632 specifically promoted the extension of *microtubule based* neuritic and filopodia-like projections in growth cones (Sayyad et al., 2015; Rösner et al., 2007). Together with our data, these results support the notion that non-muscle Myosin II is a negative regulator of fine filopodia-like protrusions via modulation of the interaction between actin and microtubules in growth cones.

However, our work also reveals additional functions for non-muscle Myosin II in decreasing perimeter, increasing roundness, and decreasing contour concavity of RGC growth cones in the optic tract. This increases our understanding of Myosin II function in developing neurons and provides a foundation for future studies exploring its underlying signaling mechanisms.

Application of Blebbistatin, the non-muscle Myosin II inhibitor, also significantly increased the number of branches along RGC axon shafts in the late optic tract/tectum of whole brains from *Xenopus* embryos. This result suggests that, in addition to inhibiting filopodia formation and extension in growth cones of RGC axons, non-muscle Myosin II also negatively regulates branching of RGC axons in late tailbud stage *Xenopus laevis* embryos. Previous studies in dissociated cortical neurons demonstrated that extension of growth cone filopodia and of collateral axon branching involve similar microtubule based regulatory mechanisms where microtubules fragment and invade the newly developing filopodia or branches (Kalil et al., 2000). Our finding that non muscle Myosin II negatively regulates both growth cone filopodia like extensions and axon shaft branches in developing RGCs *in situ* suggests that formation of growth cone filopodia and lateral axon branches in developing RGCs in *Xenopus* embryos may also rely on similar actomyosin and/or microtubule regulatory mechanisms described in the preceeding paragraph.

## Conclusion

We applied novel morphometric measurements to show that β-catenin-α-catenin and actomyosin signaling differentially regulate growth cone contours, and axon undulation and branching in RGCs within the optic tract of *Xenopus laevis* tadpoles. These findings utilize an innovative assessment of growth cone morphology, and advance our understanding of the specific mehanisms of these two essential cyto-mechanical cues in regulating formation of neuronal circuits in the native embryonic brain environment. To our knowledge, our previous publication (Elul et al., 2022) is the only prior manuscript to describe the role of β-catenin in growth cones in any developing neuronal system. Therefore, the data presented here significantly advance our scientific understanding of Cadherin adhesive signaling in regulating growth cone morphology, and associated axon pathfinding behaviors *in situ*. As described above, one possibility is that β-catenin binding to α-catenin promotes growth cone filopodia formation and contour complexity by enhancing actin polymerization. In contrast, Myosin II through its interaction with actin and regulation of actin retrograde flow, appears to normally suppress the extension of microtubules into filopodia-like protrusions (Sayyad et al., 2015; Rösner et al., 2007). Taken together, these data suggest a coordinated role for Cadherin mediated adhesion and actin cytoskeletal regulators in shaping growth cone protrusions and guiding axon pathfinding in developing neurons *in situ*. Beyond identifying core cyto-mechanical mechanisms of neuronal circuit formation, our findings may also help clarify how disruptions in these pathways contribute to cognitive, behavioral and structural defects observed in human neurodevelopmental disorders linked to mutations in Cadherin, β-catenin and Myosin II (Lee et al., 2022; Accogli et al., 2019; Wickham et al., 2019). For example, *de novo* heterozygous mutations, both missense and frameshift, in the human N-cadherin (CDH2) gene have been identified in individuals with a syndromic neurodevelopmental disorder characterized by intellectual disability and corpus callosum agenesis or hypoplasia (Accogli et al., 2019). The missense mutations impaired cell–cell adhesion in culture, whereas the frameshift mutations were predicted to truncate the β-catenin binding domain, disrupting the cadherin-actin link, mimicking the mechanism of our β-catNTERM mutant. Similarly, mutations in CTNNB1 (encoding β-catenin) are well established in neurodevelopmental disorders such as NEDSDV, which presents with intellectual disability, developmental delay, spastic diplegia, visual defects and autistic characteristics (Lee et al., 2022). A recent cohort study identified CTNNB1 mutations affecting both adhesive and regulatory domains, with associated features ranging from motor dysfunction to autism spectrum traits (Lee et al., 2022). While less studied, mutations in MYH10, encoding 1 of the 3 human non-muscle Myosin II isoforms, have also been linked to neurodevelopmental phenotypes. In 16 individuals, a range of MYH10 variants (missense, frameshift, and in-frame duplications) were associated with developmental delay, intellectual disability, autism spectrum disorder, and corpus callosum agenesis (Holtz et al., 2022).

## Data Availability

The original contributions presented in the study are publicly available. This data can be found here: Elul, Tamira (2025), “BetacateninMyosinIIGrowthConePaper”, Mendeley Data, V1, doi: https://dx.doi.org/10.17632/xpwwwwgnxs.1.
